# Effect on horizontal pressure in steel silos evoked by a sudden change in the ambient temperature

**DOI:** 10.1016/j.heliyon.2019.e01611

**Published:** 2019-05-10

**Authors:** Jakub Marcinowski

**Affiliations:** University of Zielona Góra, Faculty of Civil Engineering, Architecture and Environmental Engineering, Poland

**Keywords:** Agriculture, Civil engineering, Industrial engineering, Mechanical engineering, Structural engineering

## Abstract

The Eurocode EN 1991-4 includes provisions which refer to the stress increase in structural members evoked by the temperature differential between the silo wall and the stored material. This Eurocode presents in the clause 5.6 the formula on increase of horizontal pressure as a result of the temperature differential between the silo wall and the stored material. The derivation of this formula by two different approaches was presented in this work. Theoretical considerations were exemplified by chosen examples showing the effort increase of the silo wall as a result of a sudden cooling of the silo wall. Silos of different slenderness and of different coefficients of wall friction were considered. The performed analysis has revealed that in every case the formula based on the plane strain assumption gave higher values of the horizontal pressure increase. Calculations have revealed that the sudden temperature drop of the silo wall by 20 °C can lead even to the 35 % increase of horizontal pressure. Results of analytical solutions were supplemented by numerical simulations which have confirmed correctness of the formulae derived in this work. The alternative formula based on the plane strain assumption should replace the recommended in Eurocode formula based on the plane stress case.

## 1 Introduction

**Fig. 1:**
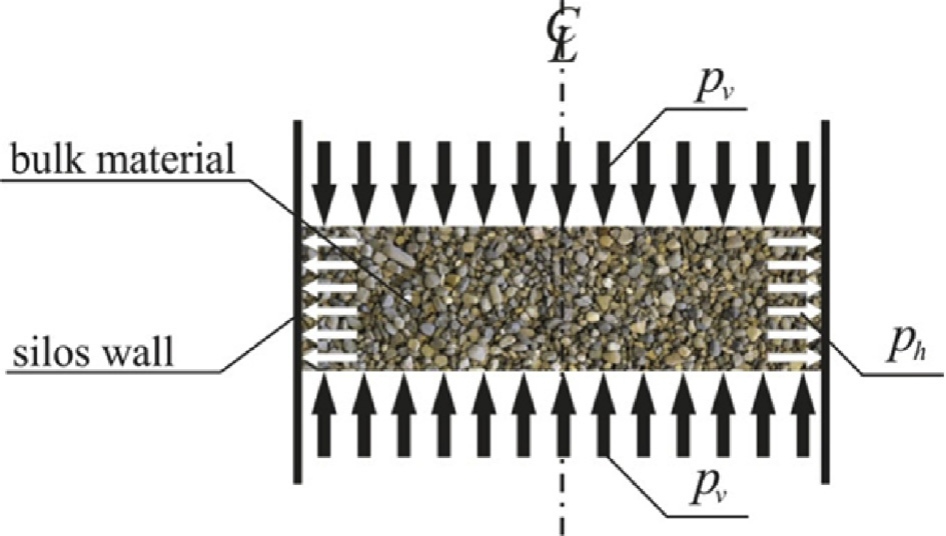
The pressure distribution inside the silo filled with a bulk material.

The bulk material stored in the silo induces vertical *p*_*v*_ and horizontal *p*_*h*_ pressures (cf. Fig. 1). These quantities are not constant and depend on many factors. One of the factor causing an increase in the horizontal pressure *p*_*h*_ of stored solid on the silo wall is a sudden drop in the ambient temperature. The shrinkage caused by the cooling of the silo wall filled with the stored material can lead to a significant increase in the interaction between the wall and the stored solid and in extreme cases it may cause the silo wall rupture.

The awareness of such a threat has been expressed in the relevant provisions of the existing load standard [1]. The 5.6 section of this standard contains provisions regarding the increase of stresses in structural elements of the silo caused by the temperature differential of the stored solid and the silo wall.

The phenomenon of stress increase in the silo walls due to temperature changes is the subject of many contemporary publications on both steel and reinforced concrete silos. Works [2] and [3] and the work [4] are worth mentioning in this context. Carson and Holmes in the paper [5] mention the change in temperature as an important factor affecting the state of stresses inside the silo construction and present an example of failure of a new silo after a few-week period of filling with fly ash. Expertise has revealed that the reason for the destruction of the silo were cyclical temperature fluctuations. During the day, the silo was heating up, the steel wall was expanding and the stored material was consolidating. During the night temperature drops, the shrinking silo wall came across the consolidated material and was unable to take on the original shape. The tensile, hoop stresses gradually increased up to the state of rupture of the screwed sheet joints (cf. [5]).

The work is a continuation of the discussion initiated in the author's work [6], which presents a detailed analysis of the standard recommendations for determining the increase of the horizontal pressure due to the temperature differential of the silo wall and the stored solid. In that paper the parametric analysis was carried out, the results of which showed a significant increase in horizontal pressure and thus explained the causes of possible failures even in the case of relatively small temperature gradients ΔT.

In the author's work [6], an attempt was made to verify the standard formula (5.101) from the Eurocode [1]. It was revealed that this formula was derived on the basis of a plane stress (identified below as PSs) assumption. In the present work an alternative approach was used: it was assumed that every horizontal layer of the stored solid works as a disc in the plane strain state (identified below as PSn). This assumption is more realistic and, as it will be shown, leads to higher values of horizontal pressure increase.

## 2 Theory

Eurocode [1] gives a formula for the increase of horizontal pressure in a cylindrical silo with radius *r*, filled with a material with an unloading effective elastic modulus of the stored solid $E_{sU}$ depending on the vertical pressure at a given depth *z* in a form:

(1) $$p_{hT}=\;C_{T}\;\alpha_{w}\;\Delta T\;\frac{E_{w}}{\frac{r}{t}\;+\;\left(1-\nu \right)\;\frac{E_{w}}{E_{sU}}}$$

where: *C*_*T*_ - the temperature load multiplier, *α*_*w*_ − coefficient of linear thermal expansion of the silo wall material (for steel 12×10^−6^ 1/^o^C), *ΔT* − temperature differential between the silo wall and the stored solid, *t* − silo wall thickness, *E*_*w*_ − modulus of elasticity of silo wall material, *E*_*sU*_ − the unloading effective elastic modulus of the stored solid, *ν* − Poisson's ratio of the stored solid (one can adopt *ν* = 0.3).

This formula was derived on the basis of general relations resulting from the theory of elasticity for a plane state of stress (PSs). Let's trace its derivation.

**Fig. 2:**
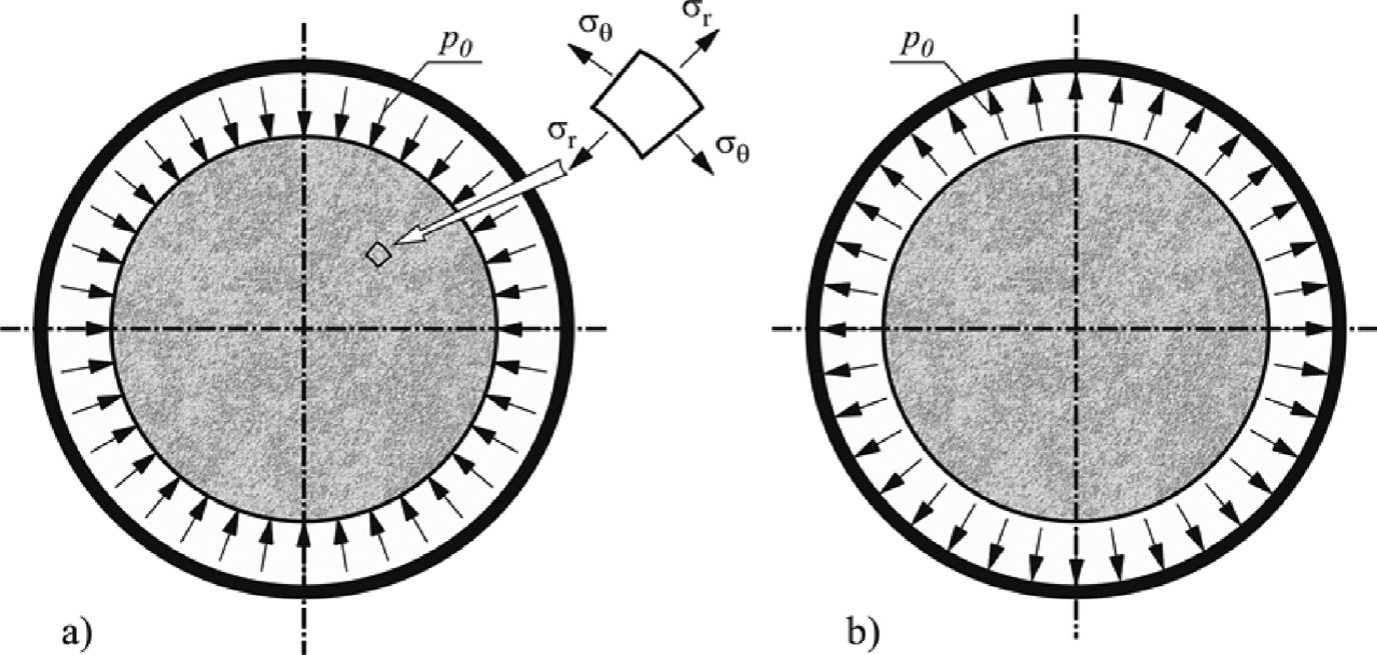
Mutual interactions of the stored material (a) and the silo wall (b).

Let as cut from the silo filled with stored solid a flat disc shown in Fig. 2. One can distinguish two continua: an elastic solid (a solid cylinder) and a surrounding sleeve (a tube). Both of these continua actually adhere to each other, and only for the purposes of the reasoning carried out here, have been separated in the drawing. The object of the search is the value of mutual impact of these continua caused by the drop in the temperature of the sleeve.

From the solution of the classic Lame problem (the problem of a thick-walled pipe, compare [7]) we obtain constant values of radial and hoop stresses in the cylindrical medium evoked by an external pressure *p*_0_ (see Fig. 2a):

(2) $$\sigma_{r}\;=\;-p_{0},\;\;\;\sigma_{\theta }\;=\;-p_{0}.\;$$

From the geometric and constitutive relations in this medium one can obtain (comp. [7]):

(3) $$\varepsilon_{\theta }\;=\;\frac{u}{r}\;,\;\;\;\;\;\varepsilon_{\theta }\;=\;\frac{1}{E}\;\left(\sigma_{\theta }\;-\;\nu \;\sigma_{r}\right)\;\;=\;\;-\frac{p_{0}}{E}\;\left(1-\nu \right)\text{,}$$

where *u* is a radial displacement, *E* and *ν* are material constants of this medium, and the constitutive relationship given in (3) is adequate to the case of a plane state of stress (PSs), which is worth emphasizing.

From the geometric relation (3) we obtain:

(4) $$u\left(r\right)\;\;=\;\;-\frac{p_{0}}{E}\;\;r\;\left(1-\nu \right)\text{,}$$

and this relationship expresses the change in the length of the radius of the solid cylinder subjected to external pressure *p*_0_.

Let us pass now to the sleeve. The circumference of the sleeve due to the exclusive operation of the temperature change (cooling *ΔT*) will take value

(5) $$L_{k}\;\;=\;\;2\;\pi \;r\;\left(1\;-\;\alpha \;\Delta T\right)\text{.}$$

If the inner surface of the sleeve is additionally influenced by the internal pressure (see Fig. 2b), then there will appear tensile hoop stresses of value

(6) $$\sigma_{\theta }\;\;=\;\;\frac{p_{0}}{t}\;\;r\;\text{,}$$

and accompanying hoop strains:

(7) $$\varepsilon_{\theta }\;\;=\;\;\frac{p_{0}}{E_{w}\;t}\;\;r\;\text{,}$$

where *E*_*w*_ is the Young's modulus of the sleeve material.

The change of the circumference of the sleeve due to the internal pressure will therefore be equal to:

(8) $$\Delta \;L\;\;=\;2\;\pi \;r\;\varepsilon_{\theta }\;=\;2\;\pi \;r\frac{p_{0}}{E_{w}\;t}\;\;r\;\text{.}$$

The total length of the sleeve circumference subjected to cooling and to internal pressure action will be equal

(9) $$2\;\pi \;r\;\left(1-\alpha \;\Delta T\right)\;+\;\;2\;\pi \;r\frac{p_{0}}{E_{w}\;t}\;\;r\;\;=\;2\;\pi \;r_{k}\text{,}$$

where *r*_*k*_ is the final radius of the sleeve.

After a rearrangement of this relation we obtain the following expression for the change of sleeve radius:

(10) $$r\;-\;r_{k}\;\;=\;\;r\alpha \;\Delta T\;-\;\;\;r^{2}\frac{p_{0}}{E_{w}\;t}\;\;\text{.}$$

The condition of compatibility of displacements at the border of the sleeve and solid cylinder (see 4) leads to equality:

(11) $$\frac{p_{0}}{E\;}\;r\;\left(1-\nu \right)\;\;=\;\;r\alpha \;\Delta T\;-\;\;\;r^{2}\frac{p_{0}}{E_{w}\;t}\;\;\text{,}$$

from which, after relatively simple rearrangements, we get the expression on the pressure at the interface between the cylinder-sleeve contact for the PSs case:

(12) $$p_{0}=\;\;\alpha \;\Delta T\;\frac{E_{w}}{\frac{r}{t}\;+\;\left(1-\nu \right)\;\frac{E_{w}}{E}}\text{.}$$

In this formula, *E* and *ν* are the Young's modulus and Poisson's ratio of the elastic medium (stored solid) in the form of a cylinder with radius *r*, *E*_*w*_ is the Young's modulus of the sleeve limiting this medium and cooled by *ΔT*.

The derived formula for the pressure increase at the interface between the two continua differs from the standard formula (1) only by the correction factor *C*_*T*_ (see formula (5.101) from the standard [1]). The value of this coefficient depends on the method of determining the unloading effective elastic modulus *E*_*sU*_ of the stored solid. It is equal to 1.2 if the elastic modulus *E*_*sU*_ was determined on the basis of tests, or 3.0 if the elastic modulus *E*_*sU*_ was estimated on the basis of the material density value and the vertical pressure value at the analyzed depth.

The method of determining the effective modulus of elasticity is described in the standard [1] in chapter C.10. Apart from the detailed experimental procedure, the standard also proposes an indirect estimation of this quantity:

(13) $$E_{sU}\;=\;\chi \;p_{vft}$$

where: χ − the modulus contiguity coefficient, $\;p_{vft}$− vertical pressure in the material at the considered depth.

The value of the coefficient *χ* can be determined on the basis of the unit weight of the stored solid *γ* from formula (C.16) from [1]:

(14) $$\chi \;=\;7\;\gamma^{3/2}\text{,}$$

in which *γ* should be expressed in kN/m^3^.

Let us correct the presented derivation assuming that we are dealing with a plane strain state (PSn), which undoubtedly corresponds better with the state of the separated stored solid layer together with the portion of the silo wall.

The constitutive relationship given in relation (3) should be replaced in this case with an expression (see [7]):

(15) $$\;\varepsilon_{\theta }\;=\;\frac{1-\nu^{2}}{E}\;\left(\sigma_{\theta }\;-\;\frac{\nu }{1-\nu }\;\sigma_{r}\right)\text{.}$$

The next steps of the derivations will be analogous to the procedure presented above for the PSs case. There is no need to repeat them, it suffices to notice that the constitutive relationship (15) differs from relation (3) in that instead of *E*, $\frac{E}{1-\nu^{2}}$ appears, and instead of ν the expression $\frac{\nu }{1-\nu }\;$. This rule has been noticed long ago. It is practically used in many plane problems of the theory of elasticity (see [7]).

The final formula for the PSs case (see Eq. (12)) can be rewritten in the equivalent form:

(16) $$p_{0}=\;\;\alpha \;\Delta T\;\frac{E_{w}E\;t}{rE\;+\;\left(1-\nu \right)E_{w}\;t}\text{.}$$

The final formula for the PSn case can be obtained directly from formula (16) by replacing material parameters in accordance with the mentioned rule:

(17) $$p_{0}=\;\;\alpha \;\Delta T\;\frac{E_{w}\frac{E}{1-\nu^{2}}\;t}{r\frac{E}{1-\nu^{2}}\;+\;\left(1-\frac{\nu }{1-\nu }\right)E_{w}\;t}\text{.}$$

Simple transformations lead to the following final formula:

(18) $$p_{0}=\;\;\alpha \;\Delta T\;\frac{E_{w}E\;t}{rE\;+\;\left(1-2\nu \right)\left(1+\nu \right)E_{w}\;t}\text{.}$$

which should be supplemented by the multiplier *C*_*T*_

(19) $$p_{0}=\;\;C_{T}\;\alpha \;\Delta T\;\frac{E_{w}E\;t}{rE\;+\;\left(1-2\nu \right)\left(1+\nu \right)E_{w}\;t}\text{.}$$

Even a cursory analysis of formulae (16) and (18) indicates that the pressure obtained for the PSn case will be greater than that obtained for the PSs case.

## 3 Example

**Fig. 3:**
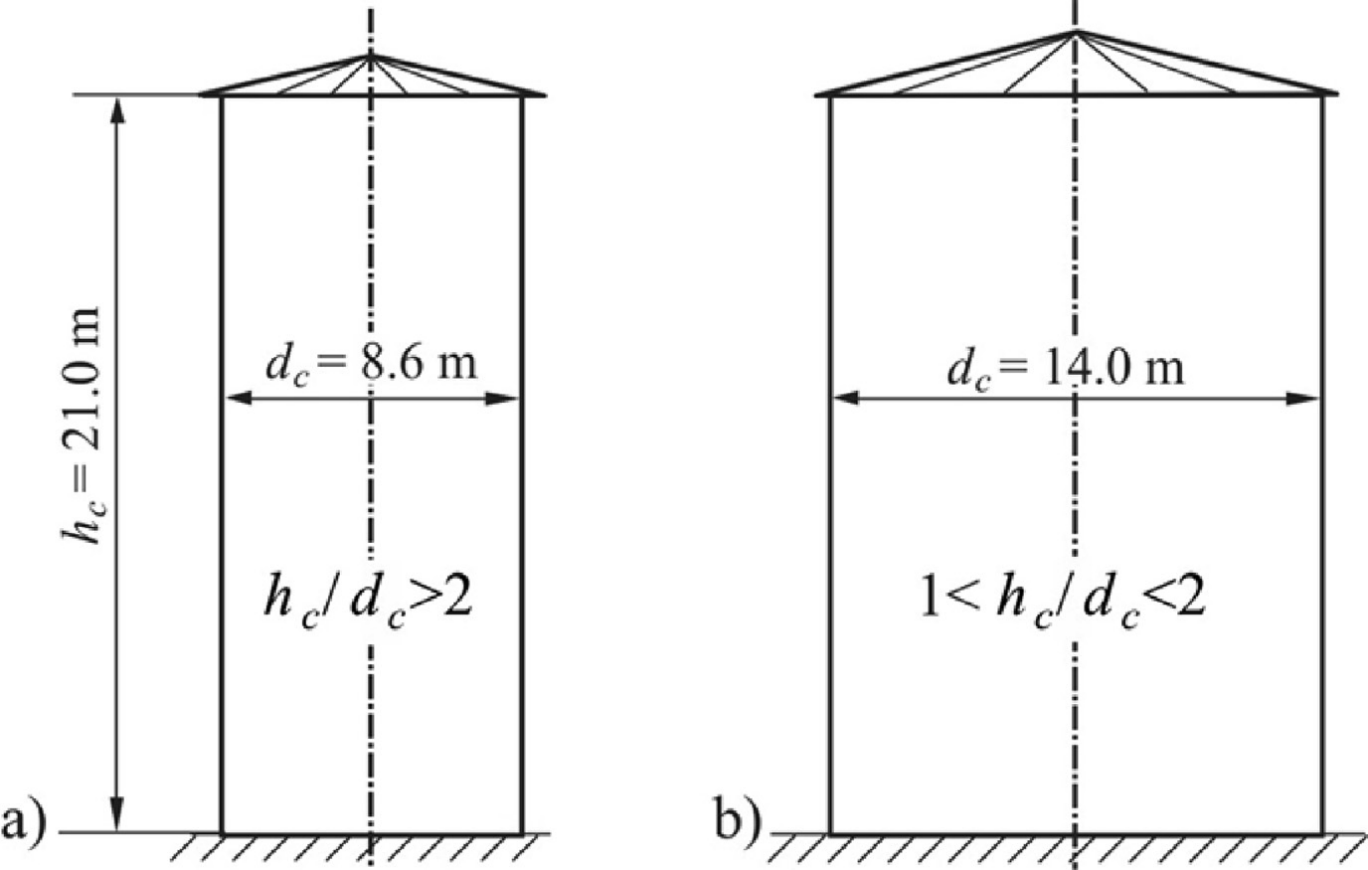
Shapes of considered silos: a) the slender silo, b) the squat silo.

To compare the values of the additional pressure caused by the temperature drop in the silo wall, let us consider flat-bottomed silos with a height *h*_*c*_ = 21.0 m and diameters *d*_c_ = 8.60 m and *d*_c_ = 14.0 m. Shapes of considered silos were shown in Fig. 3, the first one is a slender the other one - squat (see clause 3.3 in Eurocode [1]). Other data adopted for calculation: the unit weight of the stored solid (barley) *γ* = 8 kN/m^3^, lateral pressure ratio *K* = 0.59·1.11 = 0.655, coefficients of friction against the wall type *D*1, *μ* = 0.24·1.16 = 0.278 and type *D*3, *μ* = 0.48·1.16 = 0.556. These parameters were adopted on the basis of the standard [1].

Material parameters of the steel silo wall: the Young's modulus *E*_w_ = 210 × 10^6^ kN/m^3^, the Poisson's ratio *ν* = 0.3, the coefficient of linear thermal expansion of the silo wall material *α*_*w*_ = 12×10^−6^ 1/^o^C, the thickness of the steel wall at the bottom of the silo *t* = 6 mm.

In addition, the value of *C*_*T*_ = 3 in formula (1) was adopted. The same multiplier was also applied to the pressure resulting from formula (18) for the PSn case.

The values of horizontal and vertical pressures in the filling phase were determined from the standard formulae (5.1) to (5.6) from [1] (for a slender silo) and formulae (5.71) to (5.80) from [1] (for a squat silo).

The obtained values of pressures on the walls of the silo and an additional increase in pressure caused by the cooling of the steel wall by 20 °C are given in Table 1 and Table 2 for two different coefficients of friction against the wall. In the calculations both variants of the formulae (PSs and PSn cases) were used for the increase of pressure and in both the same coefficient *C*_*T*_ = 3 was used.

**Table 1:** Results for the case *μ* = 0,278 and *ΔT* = 20 °C.

Silo diameter *d*_*c*_ [m]	Unloading effective elastic modulus of stored solid *E*_*sU*_ [kPa]	Vertical stress in stored solid after filling at the bottom of silo *p*_*vf*_ [kPa]	Horizontal pressure after filling *p*_*hf*_ [kPa]	Horizontal increase in pressure due to a temperature differential, Plane stress case *p*_*hT*_ [kPa]	Increase of the horizontal pressure in %, Plane stress case	Horizontal increase in pressure due to a temperature differential, Plane strain case *p*_*hT*_ [kPa]	Increase of the horizontal pressure in %, Plane strain case
1	2	3	4	5	6	7	8
8,6	12,42·10^3^	78,44	51,37	12,05	23,45	15,91	30,96
14,0	15,68·10^3^	98,96	68,48	14,34	20,94	18,59	27,15

**Table 2:** Results for the case *μ* = 0,556 and *ΔT* = 20 °C.

Silo diameter *d*_*c*_ [m]	Unloading effective elastic modulus of stored solid *E*_*sU*_ [kPa]	Vertical stress in stored solid after filling at the bottom of silo *p*_*vf*_ [kPa]	Horizontal pressure after filling *p*_*hf*_ [kPa]	Horizontal increase in pressure due to a temperature differential, Plane stress case *p*_*hT*_ [kPa]	Increase of the horizontal pressure in %, Plane stress case	Horizontal increase in pressure due to a temperature differential, Plane strain case *p*_*hT*_ [kPa]	Increase of the horizontal pressure in %, Plane strain case
1	2	3	4	5	6	7	8
8,6	7,259·10^3^	45,83	30,01	7,21	24,03	9,59	31,97
14,0	11,87·10^3^	74,95	41,30	11,16	27,02	14,59	35,32

The results presented in Tables 1 and 2 indicate a significant increase in lateral pressure in the silos caused by wall cooling. Comparing columns 6 and 8 in Tables 1 and 2, it can be seen that the increase in the horizontal pressure for the PSn case is greater by up to 8 percentage points from the increase in the horizontal pressure for the PSs case. The maximum increase in horizontal pressure occurred in the case of the squat silo with D3 type walls and amounted to 27% and 35% for PSs and PSn, respectively.

## 4 Calculation

In order to verify the results obtained with the help of derived analytical models, numerical simulations were also performed in which the increase of horizontal pressure caused by cooling of the silo wall was analyzed. The numerical model was created in a way that guarantees full compliance with the assumptions of theoretical models used to derive presented above formulae.

**Fig. 4:**
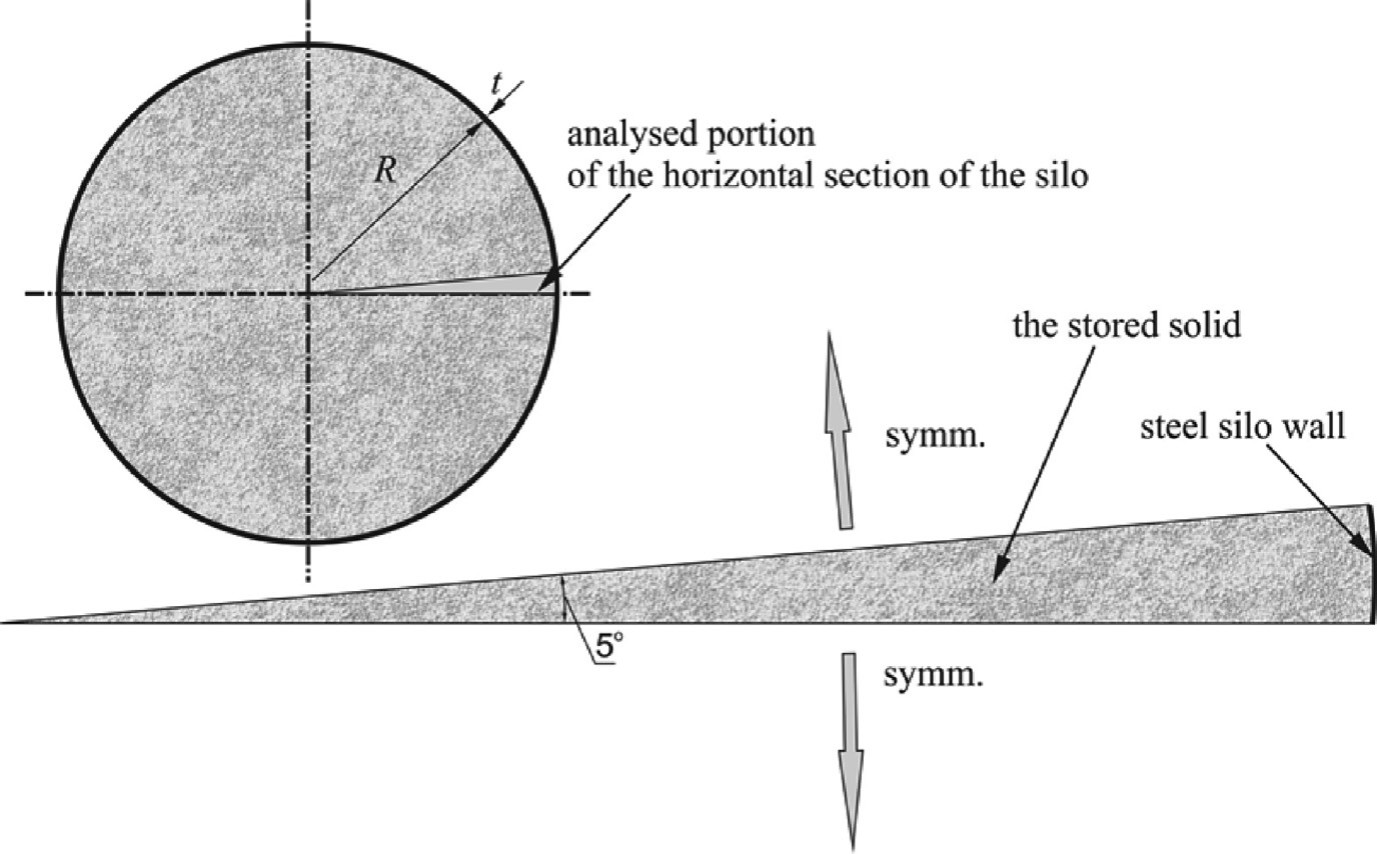
Horizontal section of the silo. Portion subjected to numerical simulation.

**Fig. 5:**
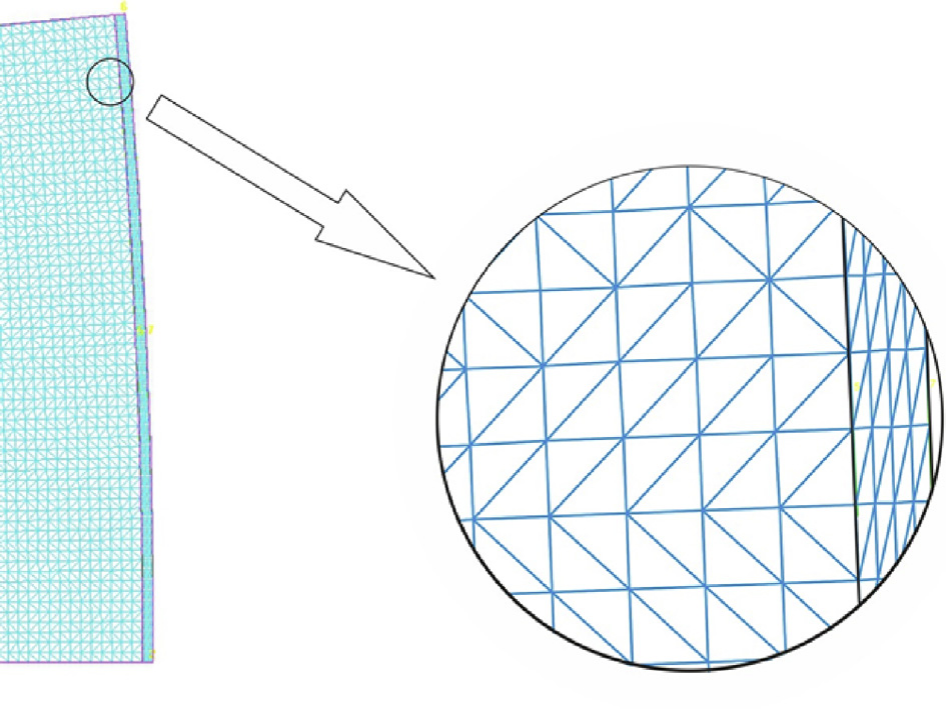
Detail of the mesh in the vicinity of the silo wall.

The stored solid layer along with the wall section was modeled numerically using the COSMOS/M program based on the finite element method (see [8]). Due to axial symmetry, only a small portion cut out from the horizontal, circular cross-section of the silo was modeled. Fig. 4 shows the considered horizontal cross-section of the silo and a portion subjected to numerical analysis. The boundary conditions reflecting the axial symmetry of the system were introduced at the edges of the cut out of the portion under consideration. Fig. 5 shows a mesh of finite elements near the silo wall. A relatively dense division into the triangular finite elements of the TRIANG family was used (see [8]). This element is used to model the problems of two-dimensional theory of elasticity, both cases of plane state of stress (PSs) and plane state of strain (PSn).

The global number of elements used to discretize the portion under consideration was equal to 53359, the global number of nodes - 27497, and this division resulted in 54864 degrees of freedom of the model. Moreover, a permanent bonding of the stored solid with the wall material was assumed, exactly as it was done in theoretical derivations. For both materials, constitutive models were adopted as for ideally elastic materials. The material parameters adopted in the calculations were identical to those in the example presented in Chapter 3.

The Young's modulus of the stored solid was adopted as the unloading effective elastic modulus determined at the silo bottom (see Table 1) *E*_*sU*_ =12.42 × 10^3^ kPa, the Poisson's ratio *ν* = 0.3. Material parameters of the silo wall: the Young's modulus *E*_*w*_ = 210 × 10^6^ kPa, the Poisson's ratio *ν* = 0, the coefficient of linear thermal expansion of the silo wall material *α*_*w*_ = 12×10^−6^ 1/^o^C, the thickness of the steel wall at the bottom of the silo *t* = 6 mm. The adopted temperature differential (cooling of the wall) was equal: *ΔT* = 20 °C. These data correspond to the slender silo of diameter *d*_*c*_ = 8.6 m and a smooth wall (D1 case) (see Table 1).

The Poisson's ratio for the wall was deliberately assumed to be zero, so that the numerical simulation exactly corresponds to the peripheral ring model adopted in the analytical derivations.

**Fig. 6:**
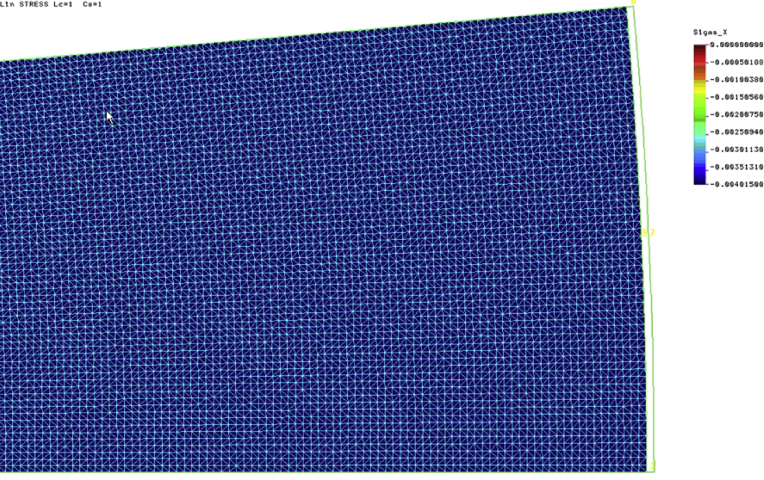
Radial stresses in the stored material. Plane stress case.

The static analysis within linearly elastic range was performed. Two cases were considered: 1. the slab in a plane stress case (PSs), 2. the slab in a plane strain case (PSn). Fig. 6 presents the distribution of radial stresses in the stored solid for the plane stress case (PSs). The value of stresses caused by lowering the wall temperature by 20 °C is equal to 4.015 kPa and is seemingly different than it results from the values given in Table 1 in the first row (case *d*_*c*_ = 8.6 m). The value *p*_*hT*_ = 12.05 kPa given in the table was obtained from analytical formulae in which the standard correction factor *C*_*T*_ = 3 was adopted. If it is neglected, then the value of *p*_*hT*_ = 12.05/3 = 4.017 kPa. This is a result almost identical to the result obtained in the numerical simulation (see Fig. 6).

**Fig. 7:**
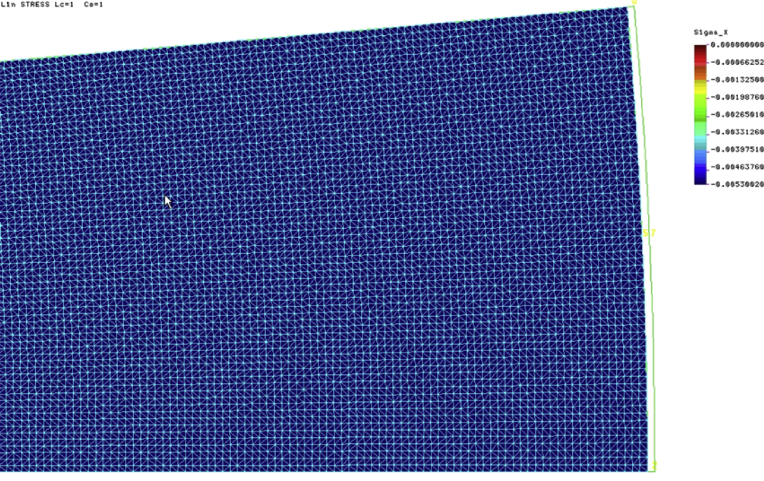
Radial stresses in the stored material. Plane strain case.

Fig. 7 presents the distribution of radial stresses in the stored solid for the plane strain case (PSn). The stress value caused by the lowering the temperature of the wall by 20 °C is equal to 5.300 kPa. The comparison with the value given in Table 1 is very good: *p*_*hT*_ = 15.91/3 = 5.303 kPa.

**Fig. 8:**
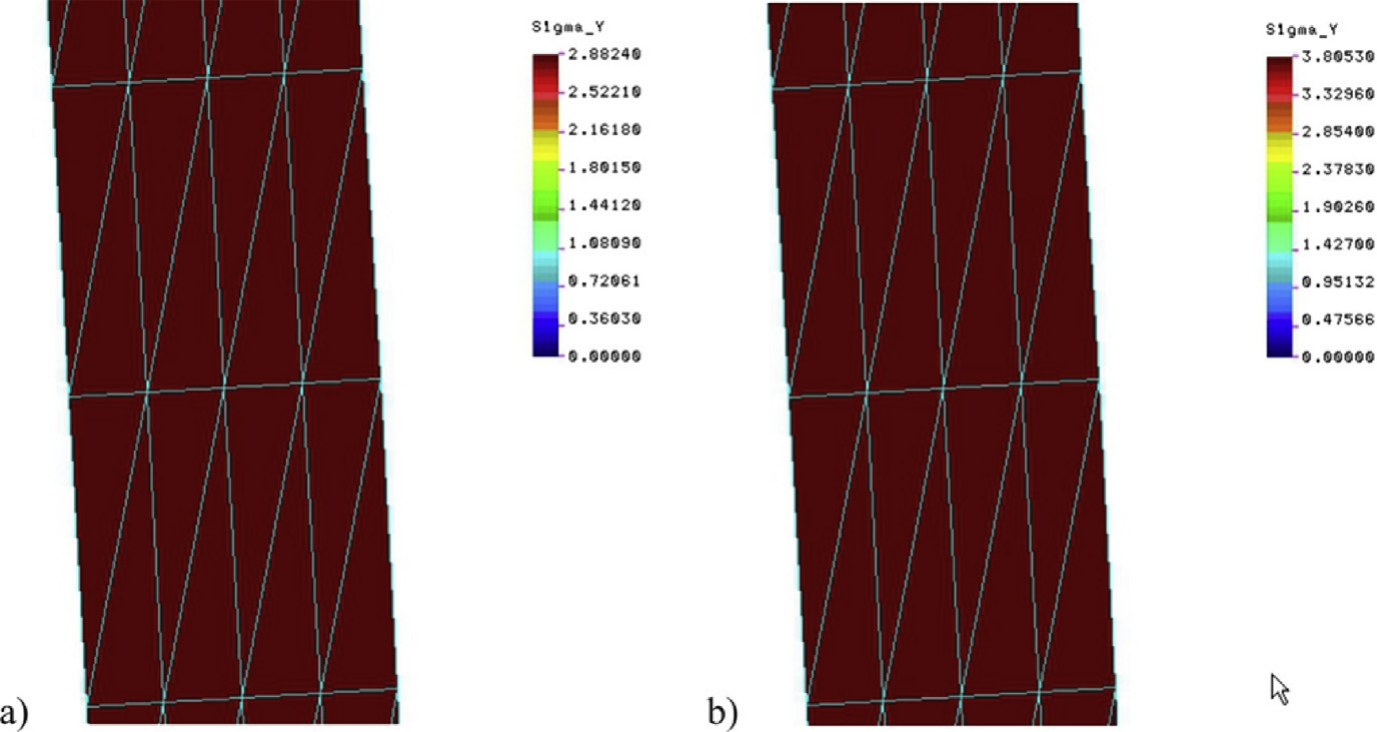
Hoop stresses in the silo wall: a) plane stress case, b) plane strain case.

Fig. 8 shows the distribution of circumferential stresses in the silo wall. They are almost constant on the thickness of a thin steel wall and amount to 2.88 MPa and 3.81 MPa for PSs and PSn cases respectively. These values almost exactly correspond to the values resulting from the theoretical relations resulting from formula (6) for horizontal pressures *p* = 4.015 kPa and *p* = 5.300 kPa, for PSs and PSn cases respectively, and namely:

(20) $$\sigma_{\theta }\;\;=\;\;\frac{p_{0}}{t}\;\;r\;=\frac{4.015}{0.006}\;4.3=2877.5\;\;\text{kPa,}$$

(21) $$\sigma_{\theta }\;\;=\;\;\frac{p_{0}}{t}\;\;r\;=\frac{5,300}{0.006}\;4.3=3798.3\;\;\text{kPa.}$$

These results are the other proof that the numerical simulations carried out fully confirm the correctness of the derived formulae on the increase in pressure caused by cooling the silo wall.

## 5 Conclusions

The principal aim of the author was the discussion of formula (5.101) inserted in EN 1991-4 [1]. It was revealed that its derivation was based on the assumption of the plane stress case. In author's opinion this formula should be replaced by the formula presented in this paper and based on the plane strain case. This state is closer the reality than the plane stress case. This new formula is also not quite adequate to the real state existing inside the silo. It possesses an important advantages: it is still simple formula, it better fits to actual conditions at the silo's bottom and it leads to safe assessment of the stress state within the silo wall.

Until now, the author could not find any experimental results regarding the increase in the horizontal pressure in the steel silo due to the temperature drop in the wall. He intends to make such measurements in the near future. Obtained results will be the basis to a verification of the formula recommended in EN 1991-4 and the present proposal.

The considerations presented in the work have concerned an in-depth analysis of the standard formula for the increase of horizontal pressure in silos caused by wall cooling. In the work, two versions of formulae were derived for the increase of horizontal pressure starting from the general relations for the two-dimensional problem of the theory of elasticity in cylindrical coordinates. The formula derived with the assumption of PSs in each horizontal layer of the silo coincides exactly with the formula recommended in the Eurocode [1]. The formula derived on the basis of PSn assumption, better models the increase of pressure on the wall and always leads to higher values, which works in favor of the silo safety.

The paper presents exemplary calculations of the pressure increase for two silos: slender and squat and for two cases of wall roughness. The increase of horizontal pressure confirmed in the calculations as a result of wall cooling by 20 °C proved to be significant. The largest increase in horizontal pressure was obtained for the case of a squat silo and walls with a high roughness. The formula derived with the assumption of PSs indicates an increase in pressure by 27%, while its equivalent resulting from PSn assumption leads to an increase of 35%. Such a significant increase in the horizontal pressure may lead to exceeding the limit state of the load bearing capacity of the silo wall and, as a result, to its failure.

The numerical simulations included in the work fully confirmed the correctness of derived analytical formulae. An analogous modeling method can be successfully applied to tanks of other shapes, also for rectangular silos, for which the Eurocode [1] does not provide analytical formulae for the increase in pressure caused by the wall temperature drop.

The design criteria should guarantee safety while meeting the condition of possibly faithful representation of physical and mechanical phenomena occurring in the described structural object. This criterion is fulfilled by formula (18) and this formula should, after complementing with the *C*_*T*_ coefficient, replace formula (5.101) in the Eurocode [1].

## Declarations

### Author contribution statement

Jakub Marcinowski: Conceived and designed the experiments; Performed the experiments; Analyzed and interpreted the data; Contributed reagents, materials, analysis tools or data; Wrote the paper.

### Funding statement

This research did not receive any specific grant from funding agencies in the public, commercial, or not-for-profit sectors.

### Competing interest statement

The authors declare no conflict of interest.

### Additional information

No additional information is available for this paper.
